# The Knockdown of USP34 Inhibits the Progression of Hepatocellular Carcinoma by Accelerating c-Myc Degradation

**DOI:** 10.5152/tjg.2025.24335

**Published:** 2025-04-21

**Authors:** Hailiang Liu, Xile Wei, Ye Nie, Jianshan Liu, Ming Fan

**Affiliations:** 1 Department of Hepatobiliary Surgery, The First Affiliated Hospital of Air Force Medical University, Shaanxi, China

**Keywords:** Aerobic glycolysis, c-Myc, hepatocellular carcinoma, ubiquitylation, USP34

## Abstract

**Background/Aims:**

The function and mechanism of ubiquitin-specific protease 34 (USP34) in hepatocellular carcinoma (HCC) were explored to provide new molecular targets for treating HCC.

**Materials and Methods:**

In the present study, bioinformatics techniques, quantitative real-time polymerase chain reaction, and western blot were used to detect the level of USP34 in HCC tissues and cell lines. Small interfering RNA of USP34 was transfected into HCC cells, and then Cell Counting Kit-8 (CCK-8) assay, wound healing assay, and transwell assay were performed to verify the proliferation, migration and invasion of HCC cells. Enzyme-linked immunosorbent assay was utilized to assess the glycolysis level in HCC cells. Co-immunoprecipitation was used to evaluate the ubiquitination level of cellular Myc (c-Myc).

**Results:**

The expression of USP34 was upregulated in HCC patients and strongly associated with worse outcomes. Meanwhile, interference with USP34 suppressed the proliferation, migration, and invasion of HCC cells. In addition, silencing of USP34 reduced the glucose uptake, lactate production and ATP content in HCC cells, as well as downregulated the expression levels of glycolysis-related proteins (hexokinase 2, glucose transporter 1, pyruvate kinase M2, and lactate dehydrogenase A). Furthermore, the knockdown of USP34 enhanced the ubiquitination level of c-Myc and increased the degradation of c-Myc. Overexpression of c-Myc reverted the inhibitory effects of si-USP34 on the malignant biological behavior in HCC cells.

**Conclusion:**

TheUSP34 regulates aerobic glycolysis and inhibits the progression of HCC by accelerating c-Myc ubiquitinated degradation.

**Main Points**

- Ubiquitin-specific protease 34 (USP34) was upregulated in hepatocellular carcinoma (HCC).
- Interfering with USP34 inhibited the proliferation, migration and invasion of HCC cells.
- Interfering with USP34 inhibited aerobic glycolysis in HCC cells.
- Interfering with USP34 promoted cellular Myc (c-Myc) ubiquitination and degradation.
- Interfering with USP34 inhibited the malignant biological behavior of HCC cells by downregulating c-Myc.

## Introduction

Hepatocellular carcinoma (HCC) is one of the most common malignant tumors in the world, whose morbidity and mortality rates are among the highest globally, posing a serious threat to the lives and health of people all over the world.^1^ Hepatocellular carcinoma patients in the early stage can be treated well by surgical resection and total liver transplantation.^2^ However, because of the insidious onset of HCC, the majority of patients are not diagnosed until the middle or late stage of the disease, losing the opportunity for radical surgical resection.^3^ Nowadays, preventing tumorigenesis at the root is the preferred strategy for treating HCC, making it crucial to understand the underlying mechanisms of HCC and develop new therapeutic approaches for HCC.

Many cells and factors are involved in the development of HCC, and the proto-oncogene cellular Myc (c-Myc) has been widely shown to be a key regulator of the malignant transformation process in the early stages of HCC.^4^ The c-Myc belongs to the nucleoprotein genes, which is considered to be one of the major factors associated with cancer.^5^ It has been mentioned in several studies that c-Myc can regulate cancer cell proliferation, metastasis, invasion, and drug resistance in HCC.^6^ Besides, c-Myc also regulates tumor metabolism, especially glycolysis. The c-Myc promotes the expression of glycolysis-related genes, such as hexokinase 2 (HK2), glucose transporter 1 (GLUT1), pyruvate kinase M2 (PKM2), and lactate dehydrogenase A (LDHA), as a means of enhancing glycolytic activity and facilitating glucose uptake and rapid conversion of glucose to lactate.^7^ Similarly, Xia et al^8^ also proposed that c-Myc exhibits a major role in the reprogramming of aerobic glycolysis and that its expression promotes the transcription of glycolysis-related genes.

Aerobic glycolysis, also called the Warburg effect, was observed in rats in 1930, which refers to the phenomenon that in the presence of oxygen, cancerous tissues also tend to break down glucose into lactic acid by glycolysis, rather than converting glucose to carbon dioxide and water via the tricarboxylic acid cycle.^9^ More and more evidence has indicated that aerobic glycolysis, an important component of metabolic reprogramming, plays a non-negligible role in cancer by regulating ubiquitination and deubiquitylation modifications.^10^ There have been many studies on aerobic glycolysis and HCC, and a variety of factors such as Adenosine 5‘-monophosphate (AMP)-activated protein kinase (AMPK), c-Myc, and hypoxia inducible factor-1 (HIF-1α) are involved in the regulation of metabolic reprogramming, which has an impact on the development and progression of HCC.^11^

Ubiquitin-specific proteases (USPs) are important components of the deubiquitinating enzymes (DUBs) families and can significantly affect HCC through post-translational modification processes.^12^ Previous studies have suggested that USP8, USP44, and USP39 are closely associated with the development of HCC and may regulate tumor growth through deubiquitinating functions.^13^^-^^15^ Additionally, Gu et al^16^ found that USP34 can regulate the survival of human pancreatic cancer cells, which is a potential therapeutic target for treating human pancreatic cancer. However, the specific role of USP34 in HCC has not yet been the subject of reports. The function of USP34 in the progression of HCC was explored in this research, with the aim of providing new molecular targets for treating HCC.

## Materials and Methods

### Tissue Samples

Hepatocellular carcinoma tissues and adjacent non-tumor samples were collected from 10 cases of HCC patients. In the selected group of HCC patients, none had received radiotherapy, chemotherapy, or targeted therapy before surgery. The study was reviewed and approved by the Institutional Medical Ethics Committee of the First Affiliated Hospital of Air Force Medical University (No. KY20172013-1, Date of approval: April 06, 2017). According to the Declaration of Helsinki, the patients whose tissues were used for this research provided informed consent.

### Cell Culture

The human HCC cell lines (Huh7, HepG2, HCCLM3 and MHCC97H) and human normal liver cell lines (IHHA-1) from the American Type Culture Collection (ATCC, VA, USA) were incubated in DMEM (Gibco, China) at 37°C with 5% CO_2_.

### Cell Transfection

The siRNA targeting USP34 (si-USP34) or control siRNA (si-NC) with scrambled sequence, and pcDNA3.1-c-Myc (ov-c-Myc) or pcDNA3.1-scramble (ov-NC) plasmids were obtained from GenePharma (Shanghai, China). MHCC97H cells were cultured until 80% confluence before transfection. The respective siRNAs or plasmids, together with Lipofectamine 2000 (Beyotime, Shanghai, China), were used for transfecting cells. The cells were harvested for subsequent study following forty-eight hours of transfection.

### Quantitative Real-Time Polymerase Chain Reaction

Total RNA was extracted from the sample using the Trizol reagent (Invitrogen, CA, USA), and its concentration was determined. The cDNA was synthesized using a reverse transcription kit (Takara, Shiga, Japan) according to the manufacturer’s protocol. Then, quantitative real-time polymerase chain reaction (RT-qPCR) was performed, and the mRNA levels of target genes were assessed by the 2^−ΔΔCt^ method with GAPDH as an internal reference. The primer sequences are as follows: USP34, forward 5’-CGT TTG GAC ATG ACG CCC TA-3’ and reverse 5’-CAT CTG CCG TTC CTG TGT GA-3’; c-Myc, forward 5’-GTC AAG AGG CGA ACA CAC AAC-3’ and reverse 5’-TTG GAC GGA CAG GAT GTA TGC-3’.

### Western Blot

Different HCC cell lines were lysed using RIPA lysate (Qiagen, NRW, Germany), and the levels of each protein were detected using the BCA kit (Thermo Fisher Scientific, MA, USA). Equal proteins were isolated by 10% SDS-PAGE and transferred to PVDF membranes (Millipore, MA, USA). Following blocking for 2 hours, the membranes were incubated with the indicated antibodies overnight at 4°C. The primary antibodies were as follows: USP34 (1:1000), HK2 (1:1000), GLUT1 (1:1000), PKM2 (1:1000), LDHA (1:1000), c-Myc (1:1000), and GAPDH (1:2000). After washing with TBST solution 3 times (Thermo Fisher Scientific, MA, USA), the membranes were incubated with secondary antibody (1:5000) for 1 hour. The results were displayed using enhanced chemiluminescence (Pierce; Thermo Fisher Scientific, Inc.).

### Co-Immunoprecipitation

The collected cells were lysed using RIPA lysis buffer (Qiagen, NRW, Germany), which releases intracellular proteins for subsequent experimental manipulation. Cell lysates were immunoprecipitated with specific primary antibodies for 3 hours at 4°C, and then incubated with protein A/G agarose beads (Thermo Fisher Scientific) for 1 hour at 4°C to ensure that the antibodies would stably bind to the target proteins. The agarose beads were repeatedly washed with wash buffer to remove non-specifically bound proteins at the end of the incubation. The resulting complexes were analyzed by immunoblotting.

### CCK-8 assay

Cell suspensions were seeded in 96-well plates and cultured in an incubator at 37°C, 5% CO_2_. The CCK-8 reagent (Dojindo, Kumamoto, Japan) was added to the wells at 10 μL per well after incubation for the corresponding time (0, 1, 2 and 3 days). The OD value at 450 nm was evaluated by a microplate reader (Bio-Rad, CA, USA) to assess cell viability. The blank well was added to the medium and CCK‑8 reagent.

### Wound Healing Assay

Cells were cultured in medium to 80%-90% confluence, and then a “clear wound” was created with a 200 μL sterile spinneret (Axygen Biosciences, CA, USA). The medium was then washed with PBS (Aladdin, Shanghai, China) to remove any cellular debris produced during the wound-making process. Subsequently, the migration was observed and recorded using a microscope.

### Cell Invasion Assay

The cells were cultured in a serum-free DMEM medium for 48 hours. Trypsin (Thermo Fisher Scientific, MA, USA) was utilized to digest cells and prepare cell suspensions. The transwell unit was properly installed to ensure a tight seal between the upper and lower chambers. The cell suspension was added to the upper chamber of the transwell, making sure that the cells were evenly distributed in the upper chamber, and the medium containing serum was added to the lower chamber. After 24 hours, migrating cells in the lower chamber were fixed with formaldehyde (Aladdin, Shanghai, China) and stained with crystal violet solution for 30 minutes. The migrated cells were photographed and analyzed by light microscopy (Bio-Rad, CA, USA).

### Enzyme-Linked Immunosorbent Assay

The medium was added to the blank wells, and buffer and cell samples were added to the sample wells. Three replicate experiments were performed for each group. Then, glucose intake, lactate production and Adenosine Triphosphate (ATP) content were detected and calculated using the glucose test kit (JianCheng Bioengineering Institute, Nanjing, China), the lactate test kit (JianCheng Bioengineering Institute, Nanjing, China), and ATP test kit (Beyotime, Shanghai, China) according to the manufacturer’s protocol.

### Statistical Analysis

To ensure the reliability of the results, all experiments were replicated across a minimum of 3 distinct groups, and the findings were expressed as “mean ± S.E.M.” The *t*-test was used when comparing 2 groups. For assessing disparities among multiple groups, one-way ANOVA followed by Tukey’s post hoc test was adopted. Statistical significance was defined as *P *< .05.

## Results

### Ubiquitin-Specific Protease 34 Was Upregulated in Hepatocellular Carcinoma

**Figure 1.**
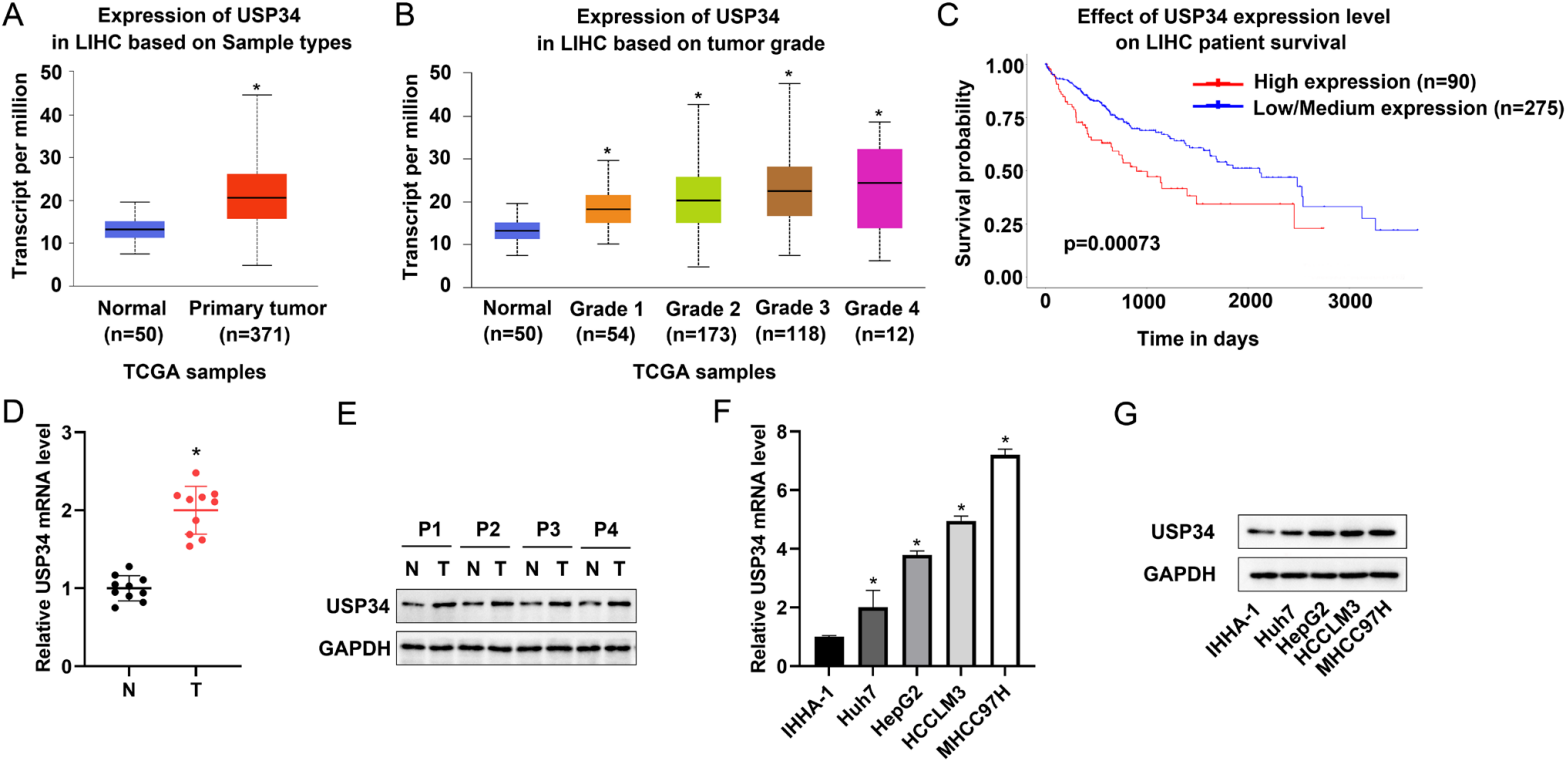
Expression of USP34 in HCC tissues and cells. The USP34 expression levels in liver hepatocellular carcinoma (LIHC) based on sample types (A) and tumor grades (B) in the UALCAN database. (C) The survival of patients with different USP34 expression levels. (D,E) The relative mRNA and protein expression of USP34 in normal tissues and HCC tissues of patients. (F,G) The relative mRNA and protein expression of USP34 in human HCC cell lines (Huh7, HepG2, HCCLM3, and MHCC97H) and human normal liver cell lines (IHHA-1). *P < .05 vs. normal tissues or IHHA-1 cells.

The expression pattern of USP34 in HCC was investigated by analyzing the level of USP34 in the UALCAN database (https://ualcan.path.uab.edu/), tissues, and cell lines. The results showed that USP34 mRNA levels were upregulated in tumor tissues compared with normal tissues (Figure 1A). Moreover, USP34 was gradually upregulated along with the pathological grade of HCC, and patients with high USP34 expression had a worse prognosis (Figure 1B and C). Meanwhile, the expression of USP34 in HCC patients’ tumor tissues and adjacent non-tumor tissues was consistent with the database. All these results confirmed that the mRNA and protein levels of USP34 in tumor tissues were markedly higher than those in adjacent non-tumor tissues (Figure 1D and E). Moreover, the mRNA and protein levels of USP34 were significantly upregulated in the human HCC cell lines (Huh7, HepG2, HCCLM3, and MHCC97H) compared to the human normal liver cell lines (IHHA-1) (Figure 1F and G). The MHCC97H cells, which had the highest levels of USP34, were selected for the subsequent studies.

### Knockdown of Ubiquitin-Specific Protease 34 Inhibited the Proliferation and Metastasis of Hepatocellular Carcinoma Cells

**Figure 2.**
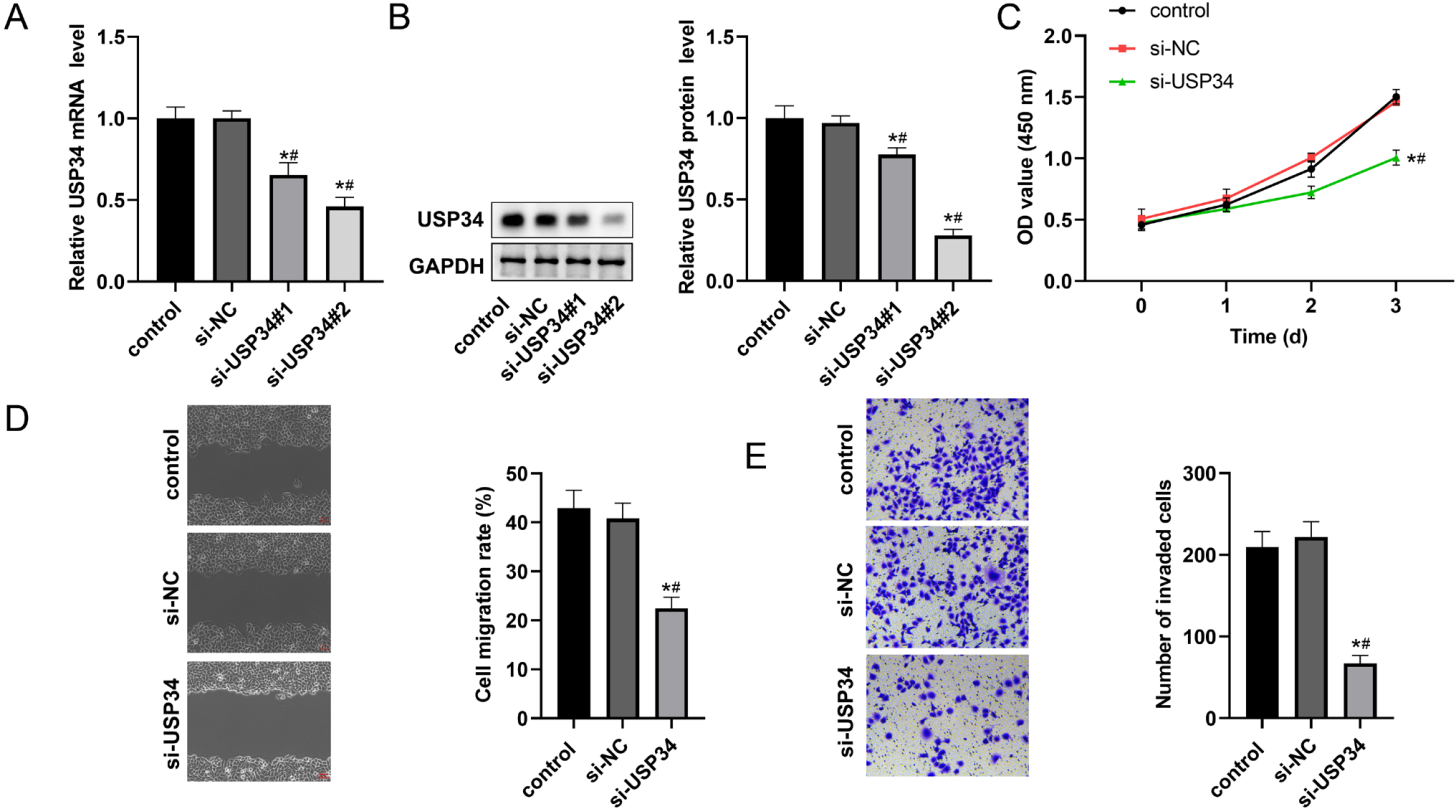
Effect of si-USP34 on HCC cell proliferation, migration, and invasion. The MHCC97H cells were transfected with si-NC, si-USP34#1, and si-USP34#2 for 48 hours. The relative mRNA (A) and protein expression (B) of USP34 in transfected cells with different small interfering RNA. The effects of si-USP34 on proliferation (C), migration (D), and invasion (E) in HCC cells. *P < .05 vs. control group; #*P* < .05 vs. si-NC group.

The relative mRNA and protein expression levels of USP34 in HCC cells were significantly reduced by transfection of si-USP34 into the cells (Figure 2A and B). In comparison with si-USP34#1, the expression of USP34 was lower in the si-USP34#2 group, so the si-USP34#2 group was subsequently used for the cell function study. As expected, the analysis revealed that knockdown of USP34 suppressed the proliferation, migration, and invasion of HCC cells (Figure 2C-E).

### Knockdown of Ubiquitin-Specific Protease 34 Inhibited Glycolysis in Hepatocellular Carcinoma Cells

**Figure 3.**
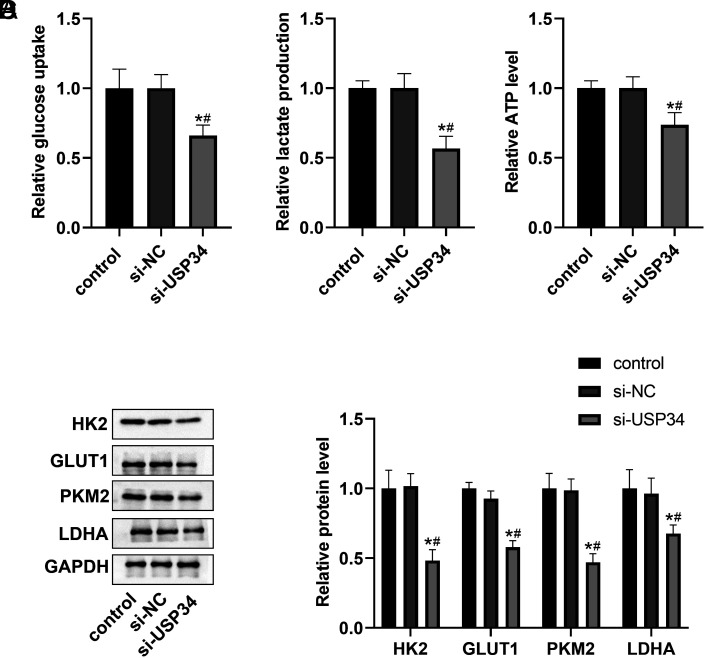
Effect of si-USP34 on glycolysis in HCC cells. The effect of si-USP34 on glucose uptake (A), lactate production (B), and ATP levels (C) in HCC cells. (D) The effect of si-USP34 on the expression of glycolysis-related proteins (HK2, GLUT1, PKM2, and LDHA). *P < .05 vs. control group; #*P* < .05 vs. si-NC group.

To clarify the role of USP34 in aerobic glycolysis, glucose uptake, lactate production, and ATP content were measured in HCC cells. As shown in Figure 3A-C, the glucose uptake, lactate production, and ATP content were markedly reduced in the si-USP34 group compared to the control and si-NC group. Furthermore, the expression of glycolysis-related proteins (HK2, GLUT1, PKM2, and LDHA) was decreased by USP34 knockdown (Figure 3D).

### Knockdown of Ubiquitin-Specific Protease 34 Promoted Cellular Myc Ubiquitination Degradation

**Figure 4.**
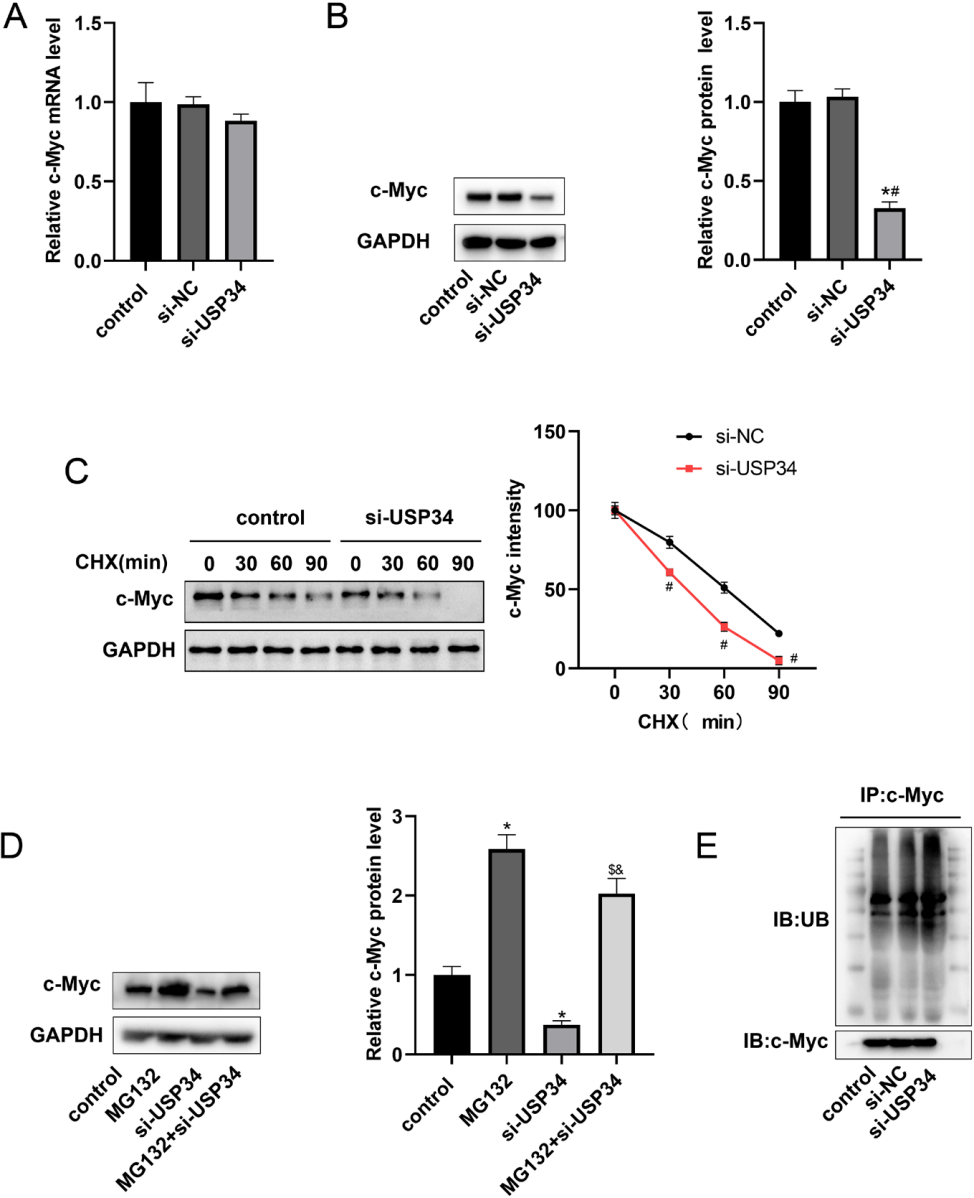
Effect of si-USP34 on c-Myc ubiquitination degradation. The effect of si-USP34 on the relative mRNA (A) and protein (B) expression of c-Myc in HCC cells. MHCC97H cells transfected with si-NC or si-USP34 were treated with the protein synthesis inhibitor CHX. (C) The effect of CHX treatment on c-Myc expression. MHCC97H cells transfected with si-USP34 were treated with MG132 (a proteasome inhibitor). (D) The effect of MG132 treatment on c-Myc expression. (E) The effect of si-USP34 on the level of c-Myc ubiquitination. *P < .05 vs. control group; #*P* < .05 vs. si-NC group; $*P* < .05 vs. MG132 group; &*P* < .05 vs. si-USP34 group.

The relative mRNA and protein levels of c-Myc were assayed, and the results revealed that the knockdown of USP34 did not affect the mRNA level of c-Myc (Figure 4A), but notably decreased the protein level of c-Myc (Figure 4B), which demonstrated that USP34 was involved in the post-transcriptional modification of c-Myc. To further clarify the regulatory mechanism of USP34 on c-Myc, CHX (a protein synthesis inhibitor) was used to treat HCC cells. It had been observed that knocking down USP34 accelerated the degradation of c-Myc (Figure 4C). Since c-Myc protein degradation is mainly regulated by the ubiquitin–proteasome pathway,^17^ it was investigated whether USP34 regulates c-Myc stability through the proteasome pathway by treating HCC cells with MG132 (a proteasome inhibitor). The data supported the fact that the c-Myc protein expression level was apparently increased after treatment with MG132 in the si-USP34 group (Figure 4D). In Figure 4E, interference with USP34 resulted in increased ubiquitination levels of c-Myc. The above findings served as evidence to support that knockdown of USP34 resulted in decreased release of ubiquitination markers of c-Myc and increased c-Myc degradation.

### Knockdown of Ubiquitin-Specific Protease 34 Suppressed the Malignant Biological Behavior of Hepatocellular Carcinoma by Downregulating Cellular Myc

**Figure 5.**
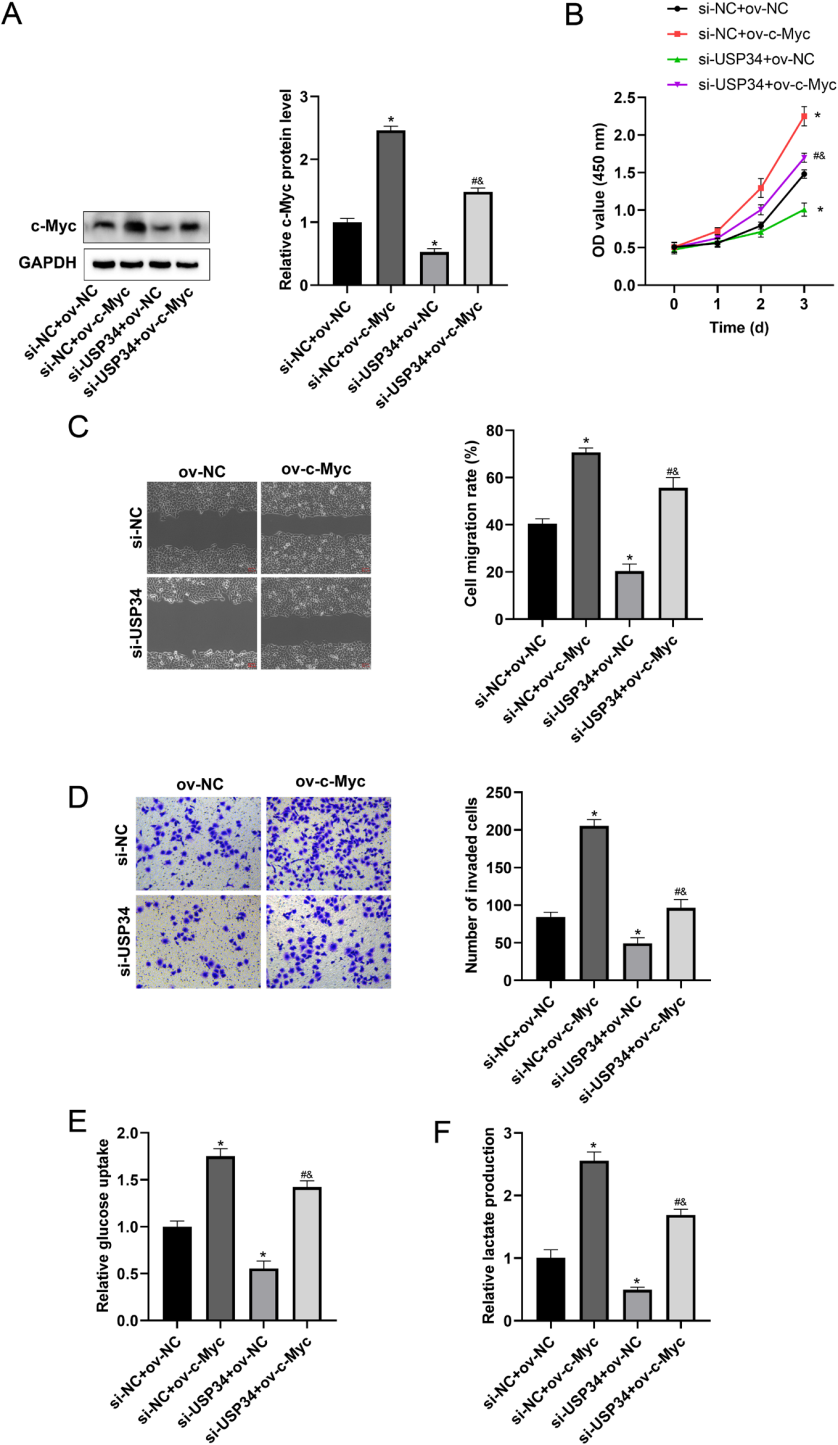
Overexpression of c-Myc reversed the inhibitory effect of si-USP34 on HCC cells. Transfected si-USP34 and ov-c-Myc alone or together into HCC cells. (A) The effect of ov-c-Myc on c-Myc expression. The effect of ov-c-Myc on proliferation (B), migration (C), and invasion (D) in HCC cells. The effect of ov-c-Myc on glucose uptake (E) and lactate production (F) in HCC cells. *P < .05 vs. si-NC + ov-NC group; #*P* < .05 vs. si-NC + ov-c-Myc group; &*P* < .05 vs. si-USP34 + ov-NC group.

To further verify the regulatory mechanism of USP34 in HCC cells, si-USP34 and ov-c-Myc were transfected into HCC cells alone or together. The results indicated that ov-c-Myc significantly promoted c-Myc expression (Figure 5A). As expected, ov-c-Myc reversed the inhibitory effect of si-USP34 on HCC cell proliferation (Figure 5B), migration (Figure 5C), and invasion (Figure 5D). Furthermore, ov-c-Myc also promoted glucose uptake (Figure 5E) and lactate production (Figure 5F), which attenuated the inhibitory effects of si-USP34 on glycolysis.

## Discussion

Ubiquitin-specific proteases are the main members of the DUB family, which play an essential role in the immunotherapy of many cancers.^18^ For example, the study by Yang et al^19^ demonstrates that inhibition of USP8 expression effectively suppresses the growth of pancreatic tumors and that mice with knockdown of USP8 have a longer survival period. Furthermore, USP8 has also been shown to be highly expressed in HCC patients, which is significantly related to bad outcomes, and the deletion of USP8 inhibits HCC cell invasion, which is expected to be a therapeutic target for HCC.^15^^,20^ In particular, USP5, as a DUB for c-Myc, increases the proliferation and metastasis of HCC cells by reprogramming the glucose metabolism.^8^ In addition to USP8 and USP5, members of the USP protein family such as USP1, USP10, USP39, and USP22 have also been implicated in the development of HCC.^21^ However, the role of USP34 in HCC remains largely unknown. In the present study, it was found that the expression of USP34 was upregulated in HCC tissues by analyzing the data from the UALCAN database. In addition, the current study further validated the expression of USP34 in HCC tissues from the hospital and different cell lines using RT-qPCR and western blot. As expected, USP34 was overexpressed in HCC tissues and cell lines. Notably, the expression of USP34 was most highly expressed in MHCC97H cells, which were characterized by high invasiveness. In general, advanced cancers were more aggressive than early cancers, which may illustrate that USP34 was correlated with the malignancy of HCC. Therefore, the expression of USP34 in different cell lines and different stage HCC tissues was consistent. Moreover, the results of functional experiments suggested that the knockdown of USP34 reduced the proliferation and metastasis of HCC cells.

Ubiquitin-specific protease 34 has been reported primarily in human pancreatic cancer, laryngeal squamous cell carcinoma, and bone formation.^16^^,^^22^^-^^24^ Currently, the specific mechanism regarding the suppressive effect of USP34 on the proliferation and metastasis of HCC cells is unknown. Aerobic glycolysis, one of the hallmarks of cancer, is regulated in HCC through multiple pathways.^11^ There are fewer studies related to USPs and tumor aerobic glycolysis. One article has proposed that METTL5-USP5-c-Myc, as a novel mechanism, can control aerobic glycolysis and promote tumor growth, providing a potential therapeutic target for treating HCC.^8^ Similarly, the present study showed that si-USP34 reduced glucose uptake and lactate production in HCC cells by examining indicators related to glycolysis. The expression levels of glycolysis-related proteins were also suppressed.

The transcription factor c-Myc, one of the products of the proto-oncogene Myc, participates in processes such as cell proliferation and metabolism, as well as regulates aerobic glycolysis in tumor cells, which is one of the key regulatory proteins of aerobic glycolysis in many tumors.^25^ In subsequent experiments, it was found that the knockdown of USP34 remarkably reduced the levels of c-Myc, suggesting that c-Myc degradation may be involved in the process of aerobic glycolysis regulated by USP34 in HCC cells. Similarly, Wang et al^6^ found that activating the Myc/LDHA axis could regulate aerobic glycolysis in HCC cells and promote tumorigenesis, Zhang et al^26^ also reported that accelerating c-Myc degradation may enhance aerobic glycolysis in HCC cells, thereby promoting cancer progression. To further verify whether USP34 relies on c-Myc-mediated aerobic glycolysis to inhibit the development of HCC, HCC cells were individually or co-transfected with si-USP34 and ov-c-Myc in this study. The results agreed with those of previous authors that overexpressing c-Myc rescued the inhibitory effects of si-USP34 on cell proliferation, metastasis, and glycolysis.^27^

The degradation of c-Myc protein in cells is mainly regulated by the ubiquitin–proteasome pathway.^17^ Studies on USP34 and c-Myc protein degradation are scarce. It has been reported that knockdown of USP34 resulted in accelerated ubiquitin-dependent degradation of gp78.^28^ The work of Pan et al^29^ suggests that knocking down USP34 in gliomas increases the level of FOXC1 ubiquitination and decreases its protein expression level. In line with the studies described above, it was also found that USP34 regulated c-Myc stability through the proteasome pathway, and silencing of USP34 increased the ubiquitination level of c-Myc and accelerated c-Myc degradation.

In conclusion, this study investigates the functionality of USP34 in HCC, revealing the key role of USP34 in the occurrence of HCC. Silencing USP34 inhibits the malignant biological behaviors and aerobic glycolysis in HCC cells by regulating the degradation of c-Myc. These findings provide us with a deeper understanding of the mechanism by which USP34 inhibits the growth of HCC through aerobic glycolysis and offers theoretical and experimental support for the use of USP34 as a molecular marker for HCC diagnosis and prognosis.

## Data Availability

The data used to support the findings of this study are available from the corresponding author upon request.
